# Amplification of the Atlantic Multidecadal Oscillation associated with the onset of the industrial-era warming

**DOI:** 10.1038/srep40861

**Published:** 2017-01-23

**Authors:** G. W. K. Moore, J. Halfar, H. Majeed, W. Adey, A. Kronz

**Affiliations:** 1 Department of Physics, University of Toronto, Toronto, Ontario Canada; 2 Department of Chemical and Physical Sciences, University of Toronto Mississauga, Mississauga, Ontario, Canada; 3 Department of Botany, MRC 164, Smithsonian Institution, Washington, DC, USA; 4 Geowissenschaftliches Zentrum, Universität Göttingen, D-37077 Göttingen, Germany

## Abstract

North Atlantic sea surface temperatures experience variability with a periodicity of 60–80 years that is known as the Atlantic Multidecadal Oscillation (AMO). It has a profound imprint on the global climate system that results in a number of high value societal impacts. However the industrial period, i.e. the middle of the 19th century onwards, contains only two full cycles of the AMO making it difficult to fully characterize this oscillation and its impact on the climate system. As a result, there is a clear need to identify paleoclimate records extending into the pre-industrial period that contain an expression of the AMO. This is especially true for extratropical marine paleoclimate proxies where such expressions are currently unavailable. Here we present an annually resolved coralline algal time series from the northwest Atlantic Ocean that exhibits multidecadal variability extending back six centuries. The time series contains a statistically significant trend towards higher values, i.e. warmer conditions, beginning in the 19th century that coincided with an increase in the time series’ multidecadal power. We argue that these changes are associated with a regional climate reorganization involving an amplification of the AMO that coincided with onset of the industrial-era warming.

Instrumental records of sea surface temperatures (SST) over the North Atlantic Ocean exhibit variability on timescales between 60–80 years that is now referred to as the Atlantic Multidecadal Oscillation or AMO^1^,^2^,^3^. Since 1900, cool periods have occurred during the 1920 s and 1980 s with warmer periods during the 1950 s and over the past few decades with a variation between the two periods in basin-averaged SST of ~0.4 °C^4^. This variability has a profound imprint on the climate system that results in a number of high value societal impacts^4^,^5^. For example between warm and cool phases of the AMO, the outflow of the Mississippi River varies by ~10%^4^. Experiments with climate models also suggest that the AMO modulates rainfall intensity in the Sahel and India as well as having an influence on Atlantic hurricane activity^6^. The spawning intensity of the Arcto-Norwegian Nordic cod has co-varied with AMO over the past 100 years^7^. More generally, the AMO has been recognized to have ecosystem impacts that include shifts in species habitat and variability in primary productivity across the North Atlantic^8^.

Climate variability on multidecadal timescales typically involves oceanic processes^3^,^9^ and there is evidence that the AMO is driven by changes in the Atlantic Meridional Overturning Circulation (AMOC), the oceanic conveyor that transports warm and salty water northwards in the upper layers of the North Atlantic with a compensatory return flow of cold water at depth^10^,^11^. However, the connection between these two modes of low-frequency climate variability is still not fully understood^12^,^13^,^14^. For example, a model generated index of the AMOC does not exhibit any power at multidecadal timescales; suggesting that the AMO and AMOC are not related^11^.

An important contributor to our uncertainty regarding the AMO stems from the fact that the industrial period, typically defined to be the middle of the 19th century onwards and roughly corresponding to the period for which widespread climate observations are available, contains only two cycles of the oscillation and as a result, there is considerable interest in identifying paleoclimate records that contain an expression of the AMO^1^,^15^,^16^,^17^. However all existing paleoclimate reconstructions that extend into the pre-industrial period are based on land-based proxies or tropical marine proxies^15^,^16^,^17^,^18^ and so there is concern that they may not fully capture variability in the AMO that has its largest amplitude and impact over the extra-tropical North Atlantic Ocean^4^,^8^,^14^.

In this paper, we introduce an annually resolved coralline algal proxy from the northwest Atlantic Ocean that exhibits multidecadal variability extending back 6 centuries. We show that this proxy contains evidence of the regional warming that began during the 19^th^ Century that was associated with an increase in the amplitude of the AMO.

## Results

**Figure 1:**
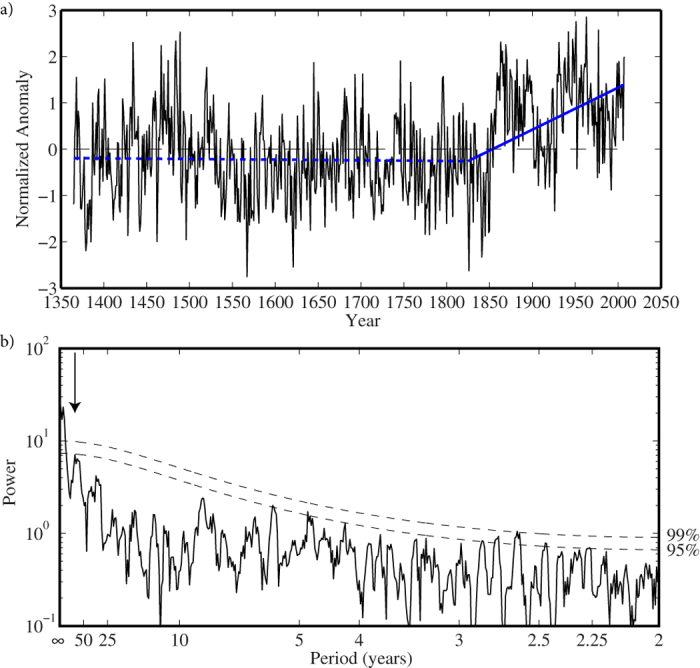
Temporal and spectral characteristics of the Labrador Sea algal time series 1365–2007. (**a**) The normalized time series (black curve). The piecewise linear least-squares fit to the time series with a breakpoint in 1825 is shown in blue. The trend post 1825 is statistically significant at the 99^th^ percentile confidence interval based on a method that takes into account the temporal autocorrelation of the time series. (**b**) The power spectra of the time series as computed by the multi-taper method with estimates of statistical significance provided by an AR(1) fit to the data. The statistically significant power at a multidecadal period of ~80 years is indicated.

**Figure 2:**
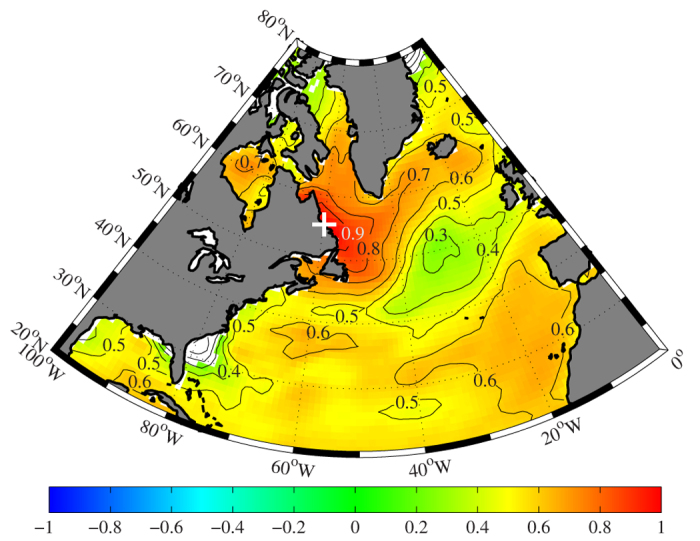
The spatial correlation in the annual mean SST from the COBEv2 dataset at the location where the coralline algal proxy was collected (indicated by the ‘+’) with the annual mean SST from the COBEv2 dataset over the North Atlantic Ocean for the period 1900–2007. The shaded regions indicate where the correlation is statistically significant at the 95th percentile confidence interval. Figure produced using MATLAB R2013b (http://www.mathworks.com).

Figure 1 shows the time series of a coralline algal proxy, hereafter referred to as the Labrador Sea algal time series^19^, from the Labrador shelf of the Northwest Atlantic Ocean for the entire period of the record 1365–2007 as well as its power spectrum calculated using the multi-taper method^20^. The collection site is under the influence of the cold inshore branch of the Labrador Current, a current that plays an important role in the surface and abyssal oceanic circulation of the North Atlantic^21^,^22^. This site contains a statistically significant signal of SST variability over the basin (Fig. 2) as well as being in a region that has warmed over the past 100 years^23^ (Supplementary Figure 1). Please refer to the Methods for more information on the time series and the spectral techniques as well as the significance tests, that take into account the red noise characteristic of geophysical time series^24^, applied to this and the other time series used in this paper.

A piecewise linear least squares fit to the time series is also shown for a breakpoint in 1825 (Fig. 1a). Breakpoints within 25 years of this date resulted in a broad minimum in the root-mean square error of the fit to the original time series. The trend after this breakpoint was statistically significant at the 99th percentile confidence interval, while the trend before it was not. The power spectrum exhibits statistically significant power at very long, i.e. centennial, timescales, consistent with the piecewise linear fit to the data, as well as multidecadal power at a period of ~80 years and inter-annual power at periods from 2.5 to 5 years (Fig. 1b).

**Figure 3:**
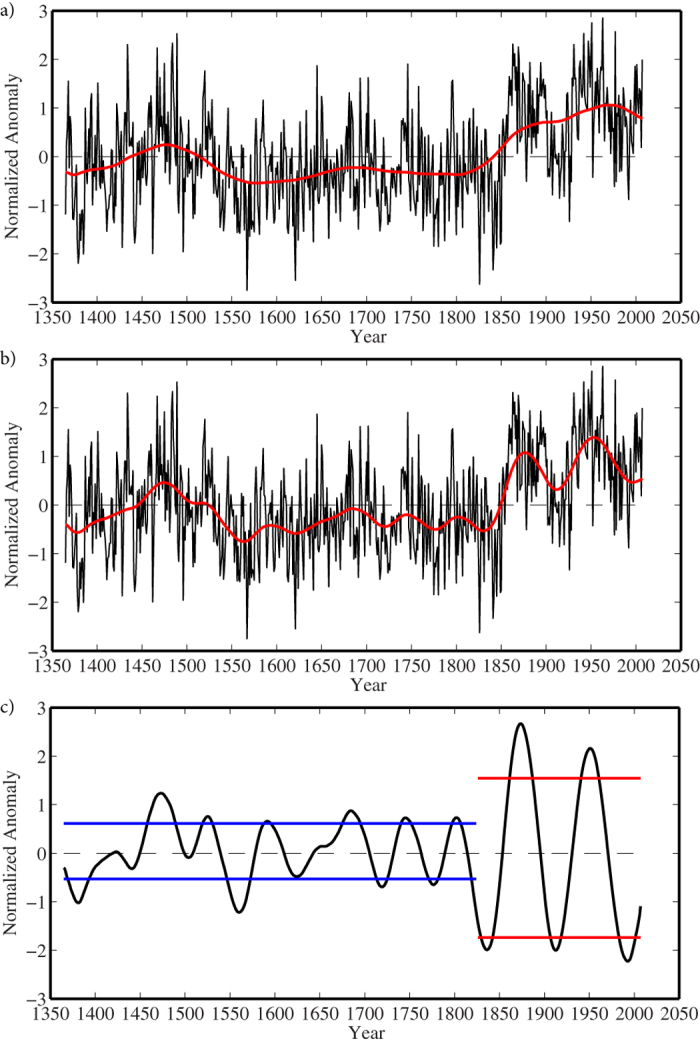
Centennial and multidecadal variability of the Labrador Sea algal time series 1365–2007. (**a**) The full time series (black curve) with the SSA reconstruction that retains variability on centennial time scales in red. (**b**) The full time series (black curve) with the SSA reconstruction that retains variability on centennial and multidecadal time scales in red. (**c**) The SSA reconstruction that retains the multidecadal mode of variability with measures of the standard deviation in this mode before and after 1825 indicated by the blue and red lines respectively.

The Singular Spectrum Analysis (SSA) technique^20^ was used to reconstruct the identified variability in the Labrador Sea algal time series on centennial and multidecadal timescales. The reconstruction that captures the centennial timescale variability explains ~25% of the variance in the time series (Fig. 3a). The mode has a local maximum during the 15th century that was followed by an ~400 year period of near constant values that ended in the early part of the 19th century after which there was a trend towards higher values. Indeed, values in the late 20th century were the highest over the 643-year long record. This behaviour is broadly consistent with the piecewise linear fit to the time series shown in Fig. 1.

**Figure 4:**
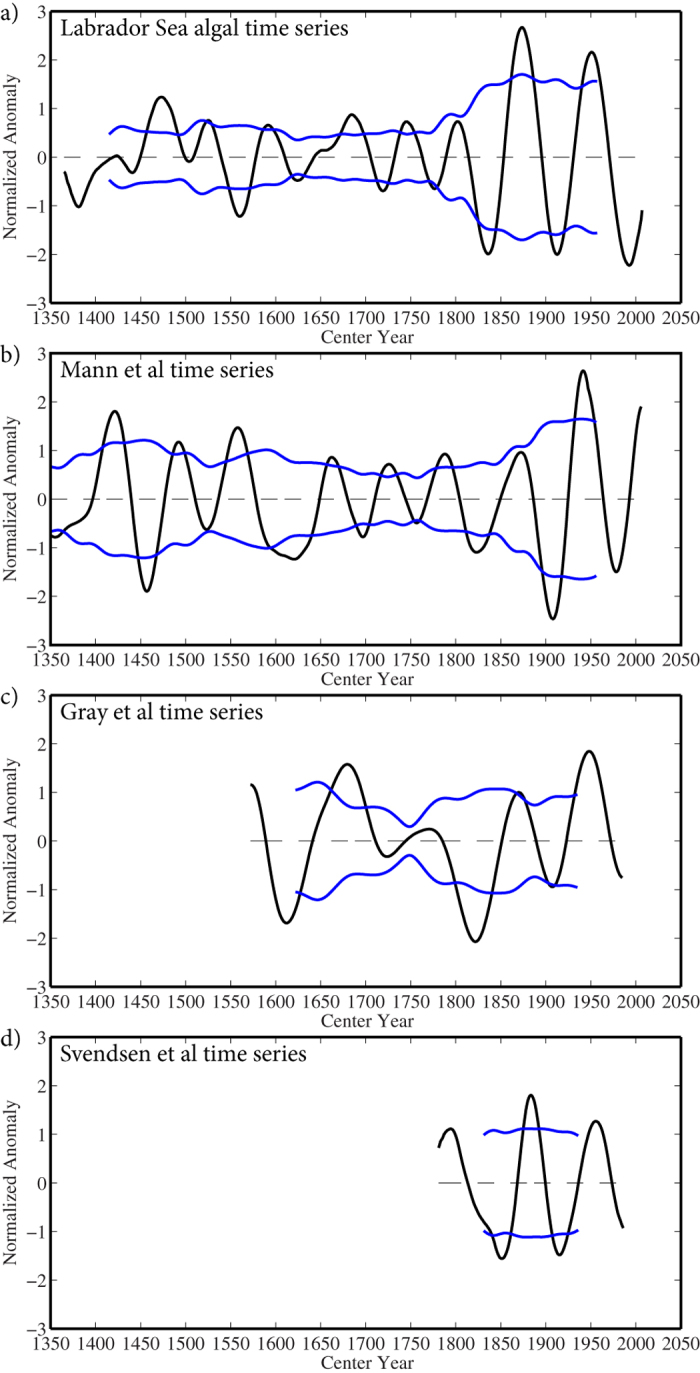
Characteristics of the multidecadal mode of variability in paleoclimate expressions of the AMO. The SSA reconstruction that retains variability on multidecadal timescales (black curves) and the 101-year moving window standard deviation (blue curves) of the: (**a**) Labrador Sea algal time series, (**b**) Mann *et al*. time series, (**c**) Gray *et al*. time series and (**d**) Svendsen *et al*. time series.

The reconstruction that retains variability on centennial and multidecadal timescales (Fig. 3b), clearly shows the modulation of the low frequency behaviour of the time series on an approximate 80 year timescale. There is also qualitative evidence for an amplification of the power at multidecadal timescales that began in the 19th century. Quantitative evidence for this amplification is provided by the reconstruction of the Labrador Sea algal time series that retains only the variability on multidecadal timescales (Fig. 3c). As one can see, there is an increase in the magnitude of the standard deviation of this mode of variability after 1825 as compared to the period before. Other breakpoints within 25 years of 1825 yielded similar results as did a moving window standard deviation calculation shown in Fig. 4.

There exist a number of paleoclimate reconstructions of the AMO that extend into the pre-industrial period^15^,^16^,^17^. The longest reconstruction, referred to as the Mann *et al*. time series, spans the period from 1300–2006 and is derived from a diverse set of screened annually resolved land-based proxy records as well as some decadally resolved marine proxies^17^. Supplementary Figure 2 shows the temporal and spectral characteristics of this time series. In agreement with the Labrador Sea algal time series, it includes a trend towards higher values beginning in the 19th century that was associated with an increase in the amplitude of the multidecadal mode of variability. However, the timing for this change occurred approximately 50 years later in this reconstruction as compared to the Labrador Sea algal time series. The moving window standard deviation calculation for this time series also shows an amplification of the power on multidecadal timescales that began during the 19th century, again somewhat later than in the Labrador Sea algal time series (Fig. 4).

Another reconstruction of the AMO, referred to as the Gray *et al*. time series, is based on 12 low pass filtered tree-ring chronologies from Europe, North America and the Middle East, all between 30°N and 70°N, and spans the period from 1572–1985^15^. Supplementary Figure 3 shows the temporal and spectral characteristics of this time series including variability on multidecadal timescales. Unlike the Labrador Sea algal and the Mann *et al*. time series, the inputs to this reconstruction were detrended and it so does not contain a signature of the warming that began during 19th century. In addition, it does not contain any evidence of an increase in the amplitude of the multidecadal variability during the 19th century (Fig. 4).

The Mann *et al*. and Gray *et al*. time series are based, for the most part, on terrestrial proxies and so there is concern that they may not fully capture variability in the AMO that has its primary expression over the North Atlantic Ocean^4^. A reconstruction that does not suffer from this possible limitation is derived from five annually-resolved coral records from the tropical North Atlantic that spans the period from 1781–1986 and is referred to as the Svendsen *et al*. time series^16^. As was the case with the Gray *et al*. time series, the inputs were detrended and so the time series does not contain evidence of the warming that has occurred since the 19th century^16^. The spectral and temporal characteristics of this time series (Supp Fig. 4) show evidence of variability on multidecadal timescales. However as was the case for the Gray *et al*. time series, there is no evidence of an amplification in the power at multidecadal timescales during the 19th century (Fig. 4).

**Figure 5:**
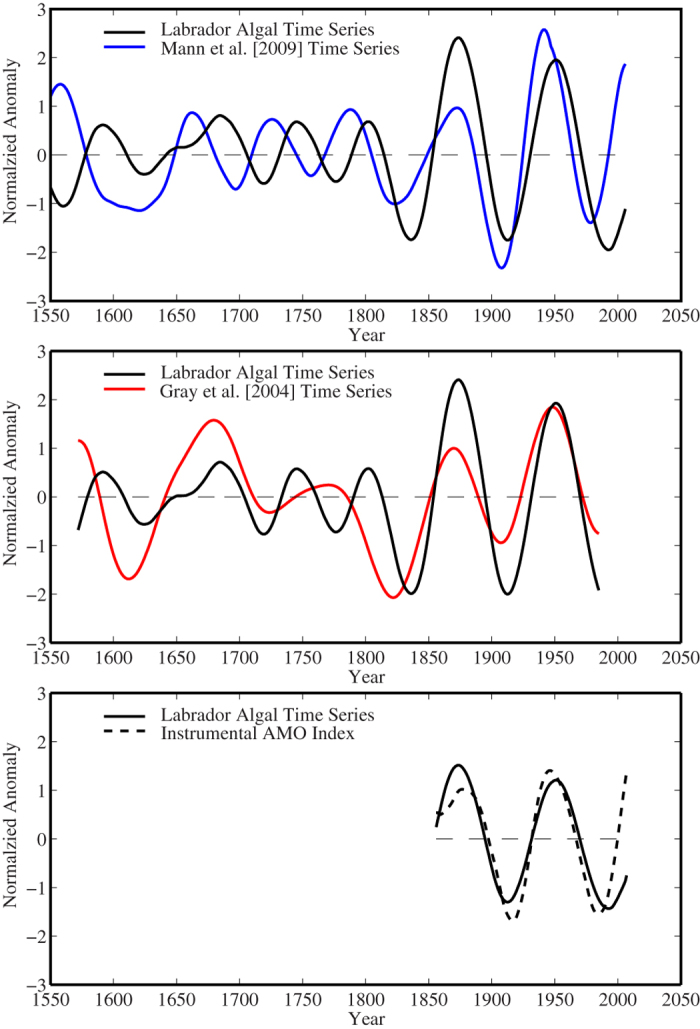
Variability on multidecadal timescales in the Labrador Sea algal time series (black), the Mann *et al*. time series (blue), the Gray et al time series (red) and the instrumental AMO Index (black dashed).

In what follows, we will restrict our attention to the 3 longest proxy records that contain a signal of the AMO, i.e. the Labrador Sea algal, the Mann *et al*. and the Gray *et al*. time series. Figure 5 examines the phase relationship between the multidecadal reconstructions for these time series and the AMO Index. Comparing the Mann *et al*. and Labrador Sea algal time series, it is clear that the former leads the later by approximately 25 years in the period prior to the 19th century after which the lead is diminished. A different behaviour is seen with respect to the multidecadal reconstructions of the Gray *et al*. and Labrador Sea algal time series where the two are uncorrelated with respect to phase before the 19th century after which they are correlated with the former leading the later by a small number of years. This tendency for an in-phase relationship during the industrial period is also found to be the case for the AMO index and the Labrador Sea algal time series.

**Figure 6:**
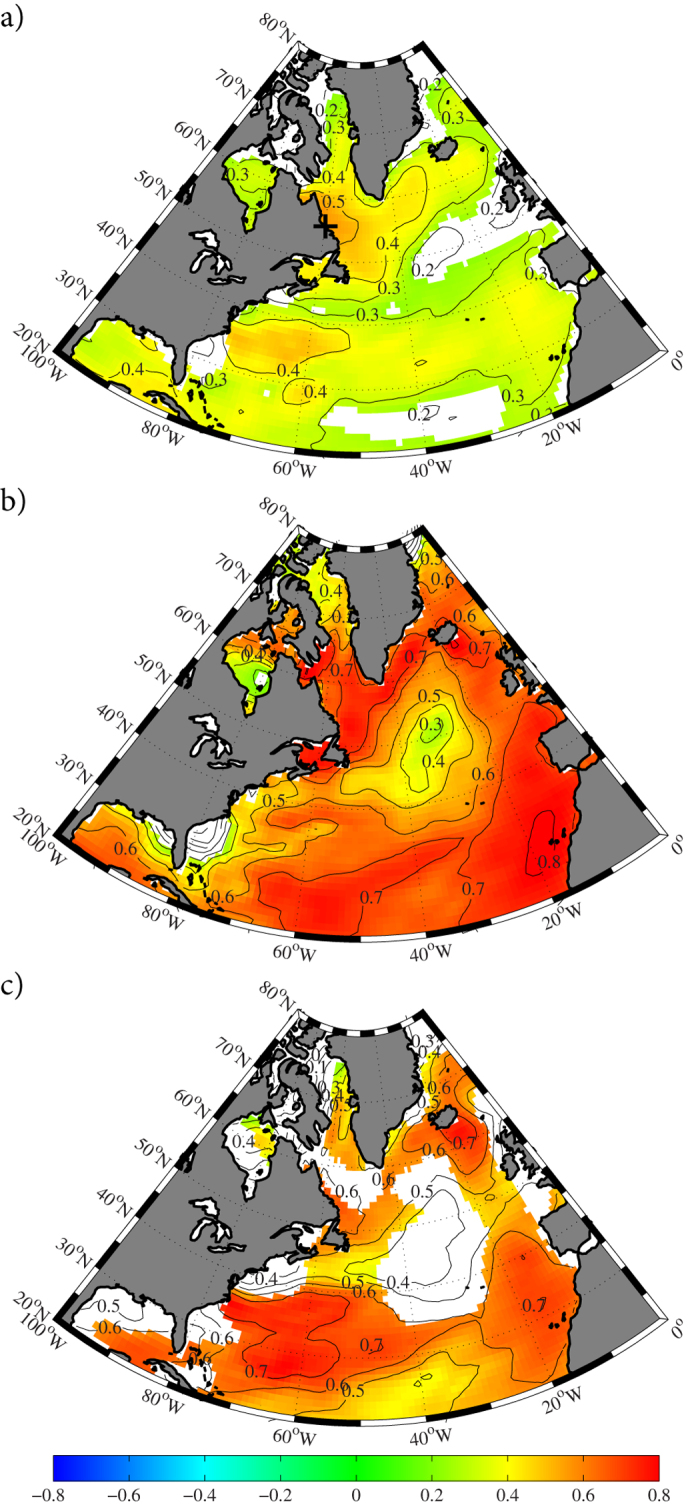
Spatial characteristics of the multidecadal variability in annual mean sea surface temperature. (**a**) The correlation of the Labrador Sea algal time series with the COBEv2 SST 1900–2007. (**b**) The correlation of the Mann et al. time series with the COBEv2 SST 1900–1985. (**c**) The correlation of the Gray et al time series with the COBEv2 SST 1900–2006. In a) the ‘+’ indicates the location where the algal samples were collected. In the shaded regions, the correlations are statistically significant at the 95th percentile confidence interval using a test that take into account the temporal autocorrelation of the SST and time series. Figure produced using MATLAB R2013b (http://www.mathworks.com).

The spatial correlation between the Labrador Sea algal, Mann *et al*. and Gray *et al*. time series and the annual mean SST from the COBEv2 dataset^25^ over the North Atlantic Ocean is illustrated in Fig. 6. Given spatial inhomogeneities of the observations in the early part of the instrumental record^16^,^25^, the correlations are shown for a period starting in 1900. All three time series have statistically significant correlations with the annual mean SST across the region. The Mann *et al*. time series was calibrated against the instrumental record and this accounts for the large magnitude of its correlation. The long-range statistically significant correlation for the Labrador Sea algal time series confirms the important role that the Labrador Current plays in the climate of the North Atlantic.

## Discussion

Both the Labrador Sea algal and Mann *et al*. time series contain evidence of a warming that occurred during the 19^th^ century. The inputs to the Gray *et al*. and Svendsen *et al*. time series were detrended and so a signature of this warming is not present. Evidence of this event is widespread in paleorecords and is referred to as the onset of the industrial-era warming^26^ that was associated with the end of the Little Ice Age^27^. This warming occurred approximately 50 years earlier in Labrador Sea algal time series as compared to the Mann *et al*. time series. The asynchronous nature of the timing of this event in paleorecords has been noted and oceanic processes have been suggested to be responsible for the differences in its timing at various locations^26^.

In addition, both Labrador Sea algal and Mann *et al*. time series are also characterized by a hitherto unrecognized increase in the amplitude of the multidecadal mode of variability during the 19th century that occurred around the same time as the respective onset of the pre-industrial warming. This increase in the amplitude of the multidecadal mode was not seen in the Gray *et al*. and Svendsen *et al*. time series. Well-defined phase lags exist amongst the various paleoclimate time series exist during the industrial period that differed in fundamental ways from those before the middle of the 19^th^ century. In particular, the Mann *et al*. time series lead the Labrador Sea algal times series by approximately 25 years prior to the 19^th^ century after which the lead was considerably smaller. The site specific nature of the Labrador time series as opposed to the global nature of the Gray *et al*. and Mann *et al*. time series suggests that the AMO may have a spatially varying non-stationary lagged character, most likely involving the ocean, that needs further clarification. The preponderance of terrestrial records in the Gray *et al*. and Mann *et al*. time series as compared to the Labrador Sea algal time series along with their differing behaviours also suggests that additional annually resolved marine records from the extratropical North Atlantic are needed to fully characterize the AMO^28^.

The Labrador Sea algal, Mann *et al*. and Gray *et al*. time series all have a minimum in the magnitude of the correlation with annual mean SST in the central North Atlantic Ocean to the southeast of Greenland. This is the region, as shown in Supplementary Figure 1, that has been previously identified as having cooled over the instrumental period^23^. Model simulations suggest that this cooling is a signature of a weakening in the AMOC^11^,^13^. The lack of correlation between the AMO proxies and the SST in this region is in agreement with this conclusion. However, there are other studies that indicate a strong link between the AMO and AMOC^10^,^29^,^30^ and the non-stationary lagged relationship between the AMO proxies identified in this paper suggests a role for the ocean in this oscillation. Clearly additional work is required to characterize the relationship between these two important modes of low-frequency climate variability.

Previous work with a marine sediment record from the Newfoundland region as well as ice core data from the Eastern Canadian Arctic indicates that the Labrador Sea and adjoining land areas underwent a warming during the middle of the 19th century^21^,^31^,^32^. Our results are in agreement with this interpretation. This warming was attributed to a large-scale reorganization of the regional climate at the end of the Little Ice Age and during the onset of the industrial-era warming that shifted the Arctic transpolar drift eastwards towards Fram Strait resulting in a reduction in the transport of cold Arctic water southwards through the Canadian Arctic Archipelago and into the Labrador Current^21^. This eastward shift in the headwaters of the Labrador Current was suggested to be associated with a change to weaker anti-cyclonic atmospheric flow over the Central Arctic Ocean^21^, a change that is consistent with a transition towards more positive values of the Arctic Oscillation (AO)^33^. This proposed transition in the AO during the 19^th^ century is in agreement with observations and climate models that suggest that more positive values of the AO are associated with a warming climate^34^,^35^.

The new information regarding the AMO that was obtained from the Labrador Sea algal time series confirm the potential that coralline algae have as a high latitude marine paleoproxy that allows for an extension of our knowledge of climate variability deep into the Holocene^18^.

## Methods

### 1) The Labrador Sea time series

Two samples (Ki1 and Ki2) of the annual increment forming long-lived coralline alga *Clathromorphum compactum* were live collected off Kingitok Island, Labrador, Canada (55.3983°N; 59.8467°W)^19^. Sample Ki1 was collected at a depth of 17 m depth and had a lifespan 1851–2011. Sample Ki2 was collected at a depth of 15 m depth and had a lifespan 1365–2011. Kingitok Island is located within the inshore branch of the Labrador Current approximately 20 km off the Labrador coast (Supplementary Figure 1). Mg/Ca ratios from the samples were measured along transects extending over the entire lifespan of each specimen using a JEOL JXA 8900 RL electron microprobe at the University of Göttingen, Germany^19^. For quantitative wavelength dispersive measurements, an acceleration voltage of 10 kV, a spot diameter of 3.5 μm, and a beam current of 12 nA were used^19^. The K-alpha signals of Mg and Ca were simultaneously analyzed for 30 s on two and three spectrometers, respectively. Samples were obtained every 15 μm along transect lines oriented perpendicular to the plane of calcite accretion. At each interval the specific subsample location was selected manually by moving the stage no more than 20 μm laterally from the transect line to avoid unsuitable sample locations (i.e., conceptacles or reproductive structures and uncalcified cell interiors)^19^. The relative mean standard deviations of multiple standard measurements were found to be no larger than 1.0% for MgO and 1.2% for CaO (2−σ). Counting statistics errors vary between 1.0 relative % and 2.9 relative % for MgO and between 0.40 relative % and 0.62 relative % for CaO (2−σ). All analyzed MgO concentrations clearly exceed the detection limit which, calculated from the background noise, was 0.015% (2−σ). Algal growth-increment widths were calculated from widths of annual Mg/Ca cycles^28^. The error associated with this calculation is +/− 15 μm, as Mg/Ca spot analyses were done at 15 μm intervals. Reconstructions were standardized to have a mean of zero and unit variance.

A combined record that is referred to in the paper as the Labrador Sea algal time series was calculated by averaging the normalized annual growth rates and annually-averaged Mg/Ca ratios, a proxy for SST^28^, from both samples^19^. Age models were established based on the pronounced seasonal cycle in algal Mg/Ca^28^. Maximum (minimum) Mg/Ca values of subannual cycles were tied to August (March), which is on average the warmest (coolest) month in the study area. Minimum Mg/Ca values represent the winter growth break. Mg/Ca time series were linearly interpolated between these anchor points using AnalySeries software^36^ to obtain an equidistant proxy time series with 12 samples per year resolution. The developed chronology was refined and thoroughly cross-checked for possible errors in the age model by comparing annual extreme values in the Mg/Ca ratio time series to imaged growth increment patterns for each individual year of algal growth. In addition, the age model was confirmed by three accelerator mass spectrometry (AMS) radiocarbon dates obtained from sample Ki2. All values (2σ calibrated radiocarbon ages) fall well within the age model derived from growth increment counting^19^. Visible inspection of the time series indicated possible anomalous behaviour in the last four years and so it was terminated at 2007.

### 2) Spectral Methods

The power spectra presented in the paper were calculated with the Multi-Taper Method (MTM), a technique that uses a set of orthogonal tapers or windows that are designed to minimize spectral leakage resulting from the finite length of the underlying time series^20^. The Singular Spectral Analysis (SSA) was used to reconstruct the centennial and multidecadal variability of the time series investigated in the paper. SSA is a technique, similar to empirical orthogonal functions employed in spatial analysis, that uses data-adaptive basis functions to separate a time series into statistically independent components that maximize the variability that each basis function describes^20^. The advantage of this technique is that it is non-parametric and therefore allows for the identification of generalized trends and complex oscillatory signals that may not be captured using conventional techniques that rely on a Fourier decomposition with trigonometric basis functions.

### 3) Statistical Significance

Time series of geophysical phenomena are often characterized by serial auto-correlation or ‘red noise’^37^. This leads to a reduction in the degrees of freedom associated with a particular time series that can have an impact on the significance of trends and correlations^38^. To take this into account, the statistical significance of the trends and correlations were assessed using a Monte-Carlo approach that generated 10,000 synthetic time series that share the same spectral characteristic as that of the underlying time series, thereby capturing any temporal autocorrelation^39^,^40^. The distribution of trends and/or correlations from the set of synthetic time series was then used to estimate the statistical significance of the actual result. With this test, the statistical significance is a function of the spectral characteristics of the time series under investigation. The Mann *et al*., Grey *at al*. and Svendsen et al time series are all the result of a low pass filter process^15^,^16^,^17^, evident from their power spectra (Supp Figs 3–5), and all have a reduction in their degrees of freedom that increases the magnitude of the correlation required to achieve a particular level of statistical significance.

### 4) The Instrumental AMO Index

The instrumental AMO Index is usually defined as the area-averaged SST over a region of the North Atlantic Ocean^4^,^41^. For the purposes of this paper, annual mean SST fields from the COBEv2 dataset^25^ were averaged over the domain from 0°N to 70°N and 75°W to 7°W, the same domain used in the original definition of the AMO Index^4^. Similar results were obtained if a domain that excludes the tropical North Atlantic was used^41^. The resulting index is available from 1850 to 2015 and was linearly detrended to remove the warming signal over this period^4^. The resulting index along with its power spectrum is shown in Supplementary Figure 5.

### 5) Relationship with the North Atlantic Oscillation

Previous work^19^ has indicated that the Labrador Sea algal time series is anti-correlated with a wintertime index of the North Atlantic Oscillation, a large scale atmospheric teleconnection represented by a pressure oscillation with centers of action near Iceland and the Azores that plays a dominant role in the climate of the region^42^. This correlation is believed to be associated with the out-of-phase relationship between sea ice extent over the Labrador Sea and the NAO^43^.

There is evidence that the low frequency variability associated with the NAO leads the AMO by ~15 years^44^. This behaviour may be associated with an oceanic response to the NAO that is integrative in nature and that can lead to multidecadal variability in SST like that associated with the AMO^45^.

## Additional Information

**How to cite this article**: Moore, G. W. K. *et al*. Amplification of the Atlantic Multidecadal Oscillation associated with the onset of the industrial-era warming. *Sci. Rep.* **7**, 40861; doi: 10.1038/srep40861 (2017).

**Publisher's note:** Springer Nature remains neutral with regard to jurisdictional claims in published maps and institutional affiliations.

## Supplementary Material

Supplementary Material
