# Supplementary material for: Amplification of the Atlantic Multidecadal Oscillation associated with the onset of the industrial-era warming
Source: Sci Rep. 2017 Jan 23;7:40861. doi: 10.1038/srep40861 (PMC5256104; doi:10.1038/srep40861)
Supplement: Supplementary Material [file srep40861-s1.pdf]

Supplementary Material

Amplification of the Atlantic Multidecadal Oscillation associated with the onset of the industrial-era warming

G.W.K. Moore, J. Halfar, H.Majeed, W. Adey, & A. Kronz

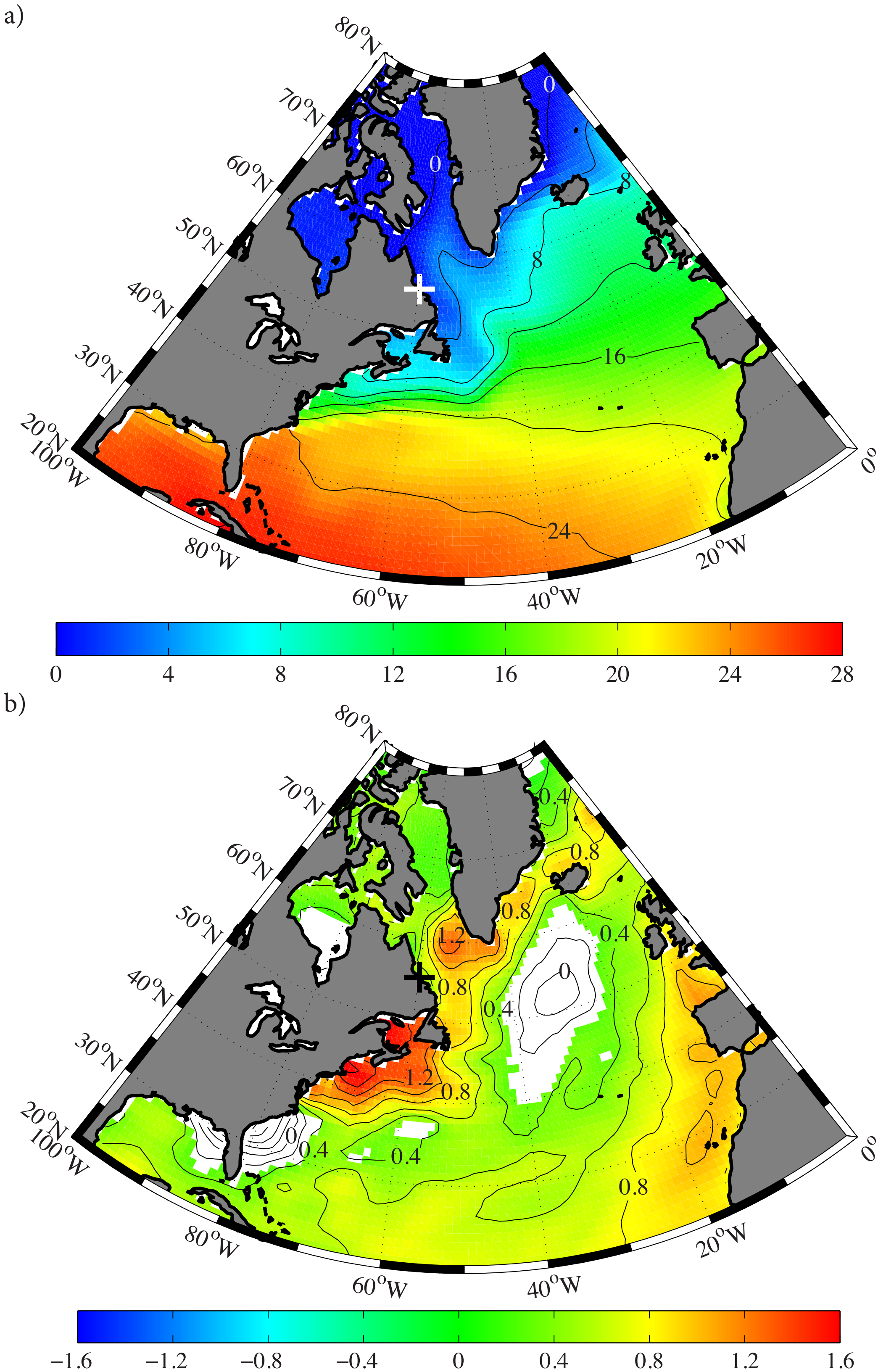

Supplementary Figure 1) Spatial variability and trend in annual mean SST from the COBEv2 dataset over the North Atlantic Ocean 1900-2014. a) The annual mean SST (°C) over the North Atlantic Ocean and b) the trend in annual mean SST (°C/decade). In b) the shaded regions indicate where the trend is statistically significant at the 95th percentile confidence interval. In a) and b), the '+' indicates the location where the coralline agae proxy was found. Figure produced using MATLAB R2013b (http://www.mathworks.com).

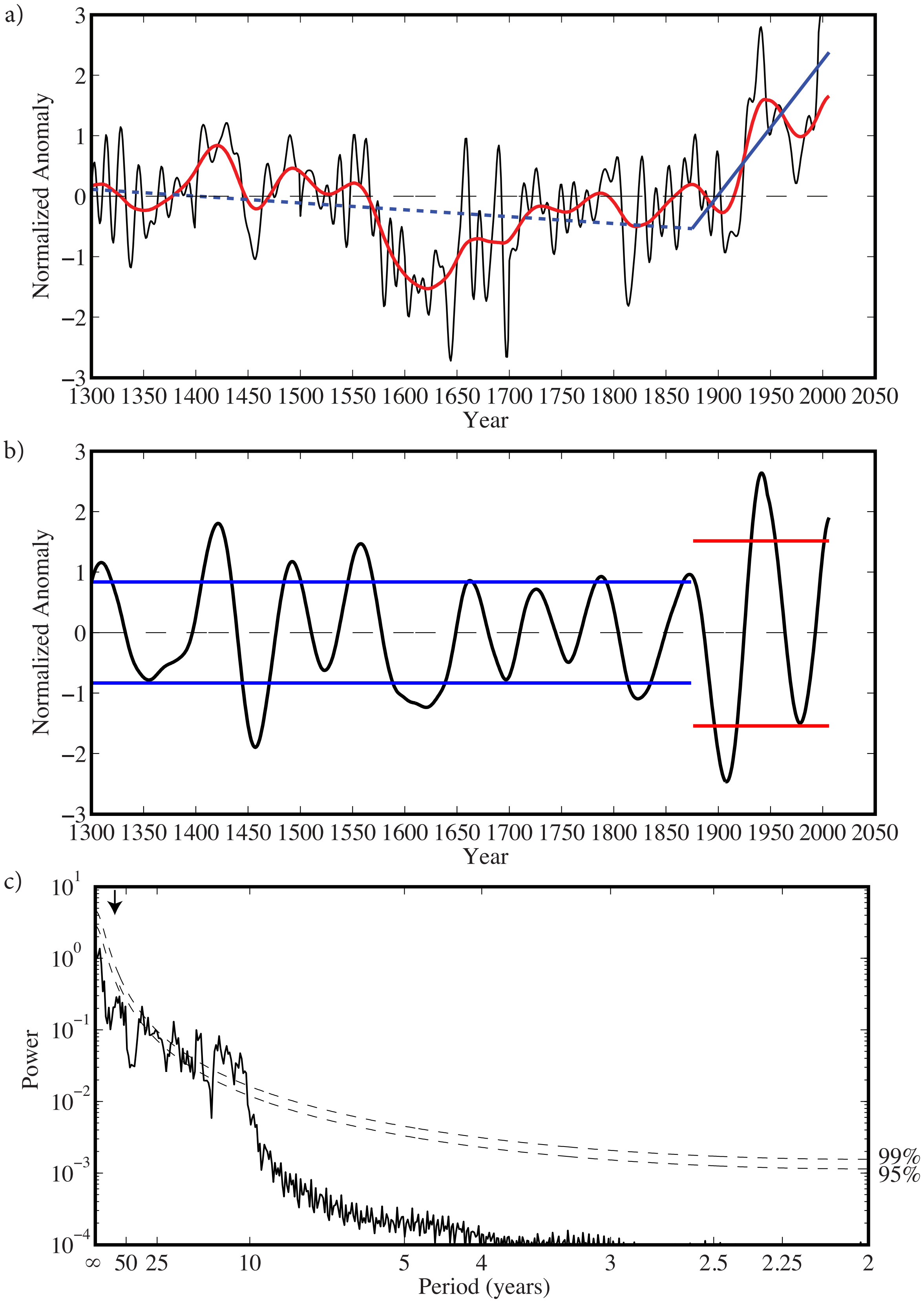

Supplementary Figure 2) Temporal and spectral characteristics of the Mann et al. time series 1300-2006. (a) The normalized time series (black curve) with the SSA reconstruction that retains variability on centennial and multidecadal time scales in red. The piecewise linear least-squares fit to the time series with a breakpoint in 1875 is shown in blue. The trend post 1875 is statistically significant at the 99th percentile confidence interval. (b) The SSA reconstruction that retains the multidecadal mode of variability with measures of the variability in this mode before and after 1875. (c) The power spectra of the time series as computed by the multi-taper method with estimates of statistical significance provided by an AR(1) fit to the data. The statistically significant power at a multi-decadal period of 80 years is indicated.

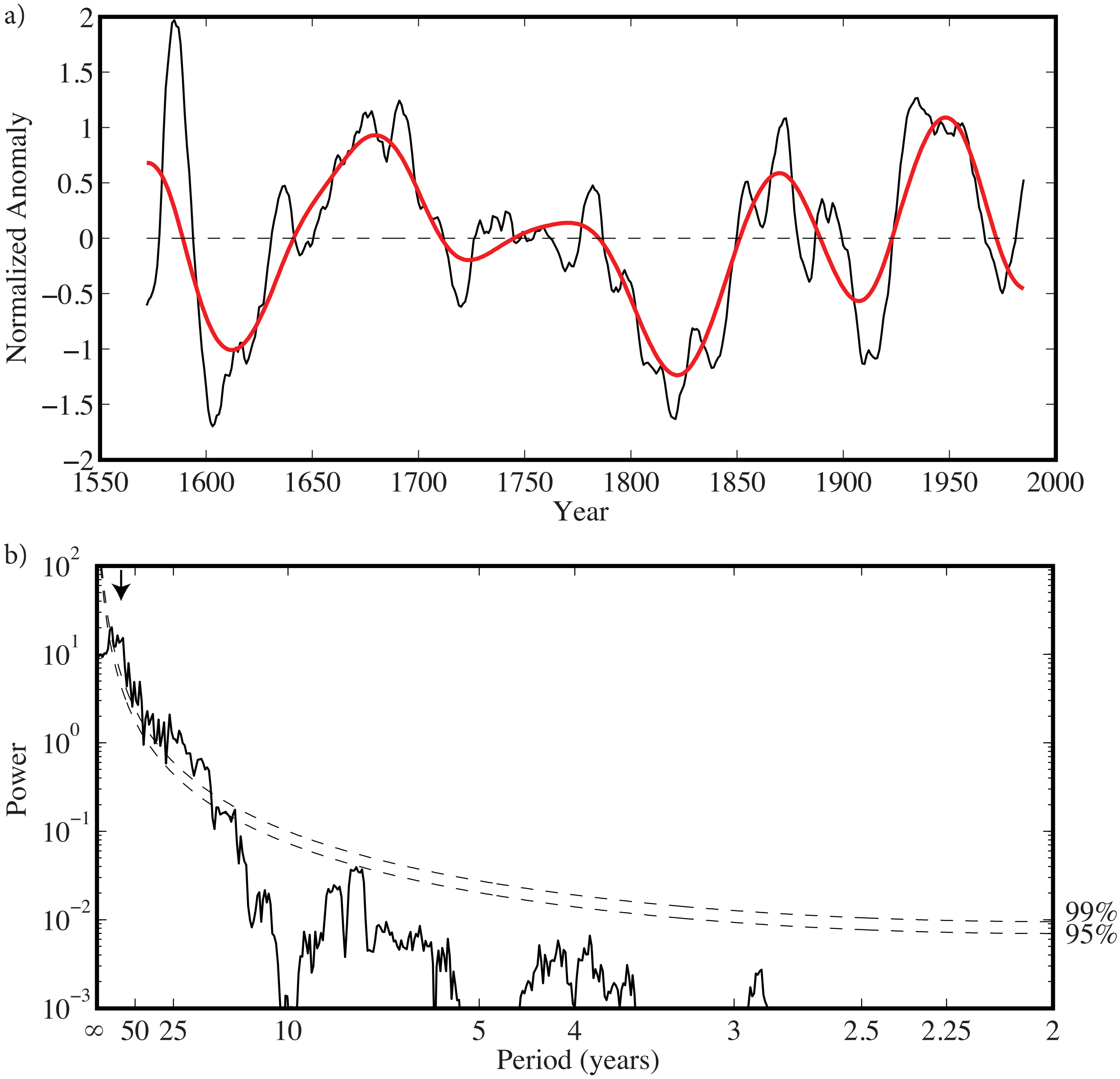

Supplementary Figure 3) Temporal and spectral characteristics of the Gray et al time series 1572-1985. (a) The normalized time series (black curve) with the SSA reconstruction that retains variability on multi-decadal time scales in red.   (b) The power spectra of the time series as computed by the multi-taper method with estimates of statistical significance provided by an AR(1) fit to the data. The statistically significant power at a multi-decadal period of 80 years is indicated.

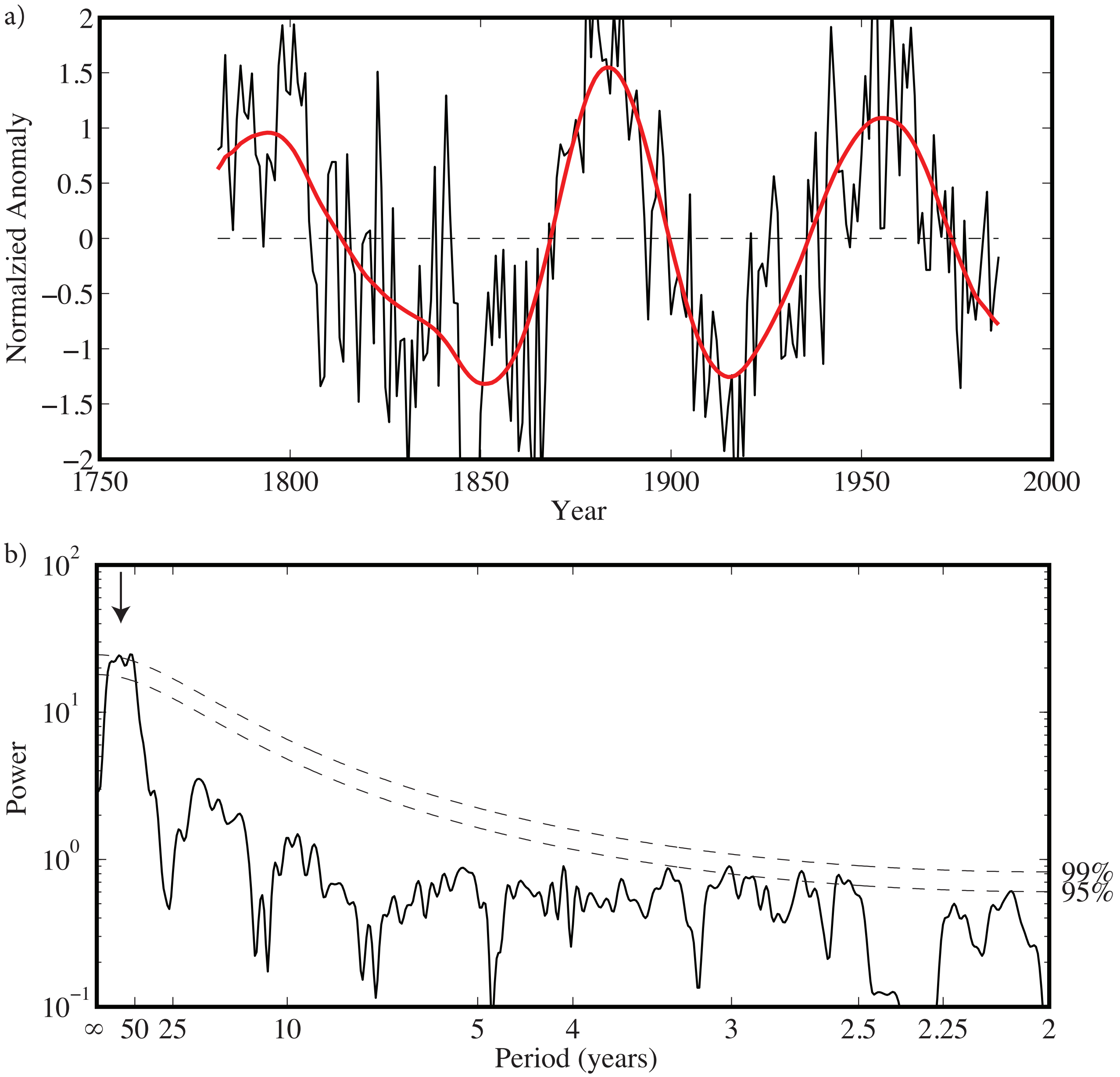

Supplementary Figure 4) Temporal and spectral characteristics of the Svendsen et al time series 1781-1986. (a) The normalized time series (black curve) with the SSA reconstruction that retains variability on multi-decadal time scales in red. (b) The power spectra of the time series as computed by the multi-taper method with estimates of statistical significance provided by an AR(1) fit to the data. The statistically significant power at a multi-decadal period of 80 years is indicated.

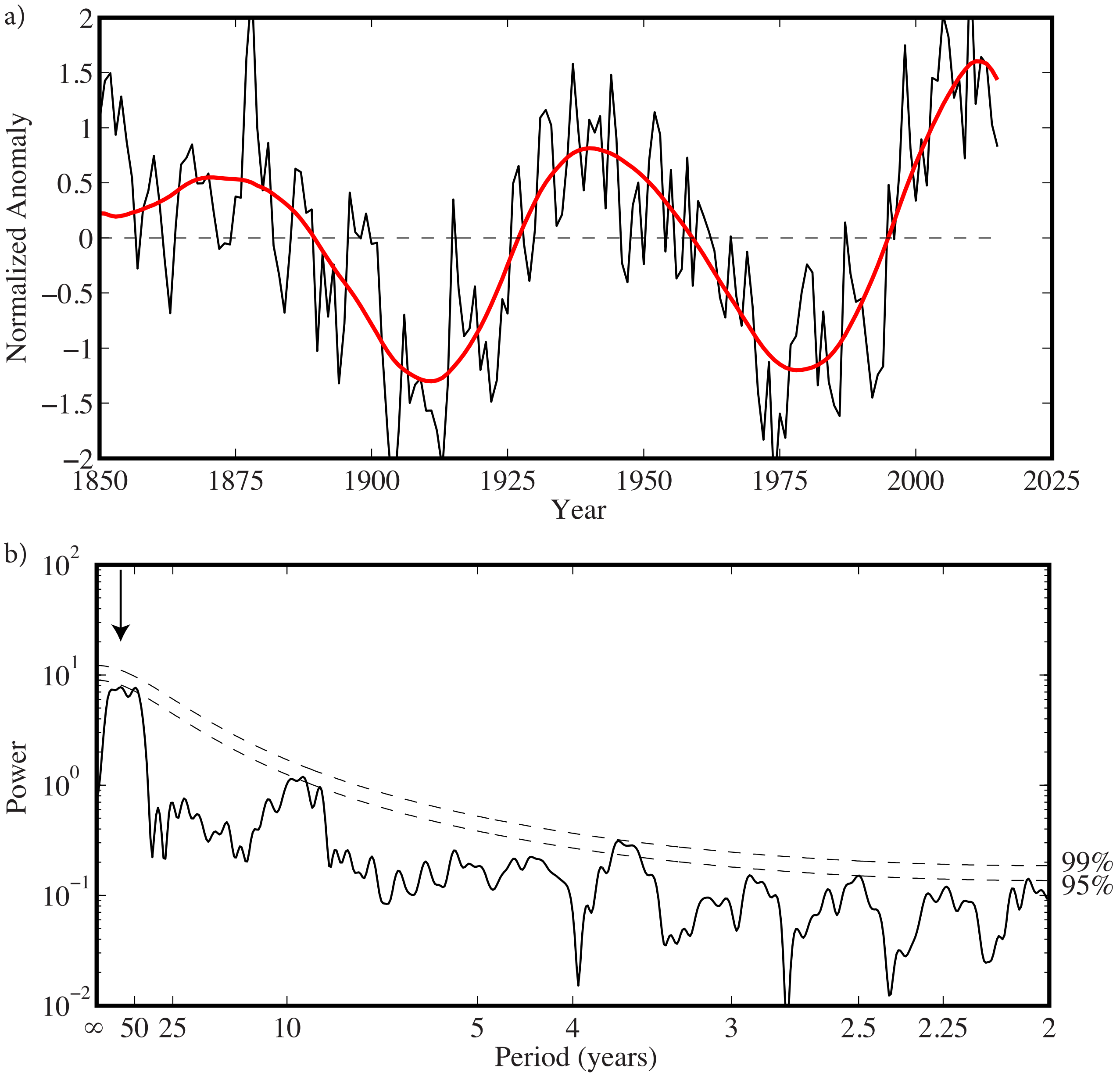

Supplementary Figure 5) Temporal and spectral characteristics of the instrumental AMO time series 1850-2015. (a) The normalized time series (black curve) with the SSA reconstruction that retains variability on multi-decadal time scales in red. (b) The power spectra of the time series as computed by the multi-taper method with estimates of statistical significance provided by an AR(1) fit to the data. The statistically significant power at a multi-decadal period of 80 years is indicated.
